# Digitization and Digital Archiving: A Practical Guide for Librarians

**DOI:** 10.5195/jmla.2022.1317

**Published:** 2022-01-01

**Authors:** April Wright

**Affiliations:** 1 adwright@hshsl.umaryland.edu, Health Sciences and Human Services Library, University of Maryland, Baltimore, Baltimore, MD

The Practical Guides for Librarians series aims to provide “authoritative, practical information and guidance on a wide spectrum of library processes and operations” [1]. *Digitization and Digital Archiving: A Practical Guide for Librarians* discusses equipment, storage, copyright, and challenges presented with digital archiving. It also includes a full chapter devoted to born-digital materials such as software, databases, websites and email. There are no case studies to illustrate best practices and lessons learned. If you are looking for a book that discusses the value and implementation of digital archiving in a social context, this is not the book for you. This book covers tools and methods and will be useful for library professionals and students who are interested in starting a digital archives project.

In the introduction, the author explains that while each chapter builds on the previous one, the book need not be read cover to cover. True to this declaration, the book is a solid introduction to digital archiving and the tools needed to do it. Although the author does not assume a base of knowledge about computers, even those adept at computers will learn something about the technology, how and why it works, and how to best leverage it for digital archiving. Throughout the text, the author effectively uses figures, tables, and textboxes to highlight comparisons and distill relevant points. The “Key Points” section at the end of each chapter makes it easy to glean information of note from the chapter along with a brief introduction to what will be covered in the next chapter.

Chapter 1 explains how digital archiving is different from traditional archiving, goals for digital archiving projects, highlights of some of the challenges of digital archiving, and issues to consider when starting a digital archive, including budget, time, scope, staffing, and partnerships. Chapter 2 provides an overview of computers and how they store information. The author then delves deeper into how computers interpret and store images, text, and audio and video in chapters 3, 4 and 5, clearly building on the information presented in chapter 2.

Chapter 6 covers born-digital data. In chapters 7–10, the author discusses the evolution of methods and media used to capture and store data, their storage capacities, the advantages and disadvantages of each, and how they should be handled and stored. Chapters 12–14 bring the reader into a more concrete explanation in creating the digital archive. Chapter 12 supplies a brief examination of equipment needs and whether to consider outsourcing. Chapters 13 and 14 explore access and copyright. Chapter 15 discusses some of the technological challenges posed by digital archiving that will need to be considered when developing policies.

Finally, chapter 16 provides guidance on creating a project plan for an archive. In a sense, it is the guide for a capstone project using all the information presented in previous chapters of the book. Previous chapters provided the beginner in digital archives with the language and basic knowledge for engaging in an archives project.

*Digitization and Digital Archiving: A Practical Guide for Librarians* would work well as a reference text. It would be a practical addition to a public or community archives library collection, library schools, and any other collection whose mission may include preservation of materials. This is a second edition, so, as new trends surface, updates to this edition will need to be made.
